# Trends in Prevalence, Treatment, and Control of Hypertension According to 40-Year-Old Life Expectancy at Prefectures in Japan from the National Health and Nutrition Surveys

**DOI:** 10.3390/nu14061219

**Published:** 2022-03-14

**Authors:** Mizuki Sata, Tomonori Okamura, Nobuo Nishi, Aya Kadota, Mieko Nakamura, Keiko Kondo, Yukiko Okami, Kaori Kitaoka, Toshiyuki Ojima, Katsushi Yoshita, Katsuyuki Miura

**Affiliations:** 1 Department of Preventive Medicine and Public Health, School of Medicine, Keio University, 35 Shinanomachi, Shinjuku-ku 160-8582, Japan; okamura@z6.keio.jp; 2 International Center for Nutrition and Information, National Institutes of Biomedical Innovation, Health and Nutrition, 1-23-1 Toyama, Shinjuku-ku 162-8636, Japan; nnishi@nibiohn.go.jp; 3 NCD Epidemiology Research Center, Shiga University of Medical Science, Seta Tsukinowa-cho, Otsu-shi 520-2192, Japan; ayakd@belle.shiga-med.ac.jp (A.K.); kon@belle.shiga-med.ac.jp (K.K.); okami@belle.shiga-med.ac.jp (Y.O.); kitaoka@belle.shiga-med.ac.jp (K.K.); miura@belle.shiga-med.ac.jp (K.M.); 4 Department of Community Health and Preventive Medicine, Hamamatsu University School of Medicine, 1-20-1 Handayama, Higashi-ku, Hamamatsu-shi 431-3192, Japan; miekons@hama-med.ac.jp (M.N.); ojima@hama-med.ac.jp (T.O.); 5 Department of Food and Human Health Sciences, Graduate School of Human Life Science, Osaka City University, 3-3-138 Sugimoto Sumiyoshi-ku, Osaka-shi 558-8585, Japan; yoshita@life.osaka-cu.ac.jp

**Keywords:** national health and nutrition survey, blood pressure, health inequality, disparity by prefecture, ecological study

## Abstract

The prevalence of hypertension has been decreasing in Japan due to improved medical treatment and a decrease in dietary salt intake. However, disparities in the prevalence, treatment, and control of hypertension are expected to occur in different regions. This study aimed to investigate the trends in the prevalence, treatment, and control of hypertension at the prefectural level of life expectancy among Japanese population. We used data from the National Health and Nutrition Survey and analysed the individual survey information of individuals aged 40–69 years by dividing it into six terms, i.e., 1995–1997, 1999–2001, 2003–2005, 2007–2009, 2012, and 2016. Prefectures were classified into four groups according to their 40-year-old life expectancy in 2000. Outcome values were standardised to the population by 10-year age groups in 2010, and they were tested by two-way analysis of variance according to six terms and life expectancies. The prevalence of hypertension tended to decrease, especially among women, whereas the treatment and control tended to improve from the first to the sixth period in both men and women. The prevalence and treatment of hypertension in men with longer life expectancy tended to be lower than that in other groups, and there was no obvious difference in the control. In women, there were no obvious differences in the prevalence, treatment, or control. Reducing the prevalence of hypertension by improving lifestyle factors, such as high salt intake in each prefecture with a relatively short life expectancy, may be important to resolve the disparity in life expectancy among prefectures.

## 1. Introduction

Hypertension is one of the most established risk factors for cardiovascular disease (CVD) [1,2,3]. Globally, the age-standardised prevalence of hypertension among people aged 18 years and older decreased from 29.5% and 26.1% in 1975 to 24.1% and 20.1% in 2015 in men and women, respectively [4]. In Japan, the mean systolic blood pressure (SBP) decreased in both men and women aged 30–79 years, whereas the treatment rate among people with hypertension and control rate among those treated people with hypertension increased substantially from 1961 to 2010 [5]. On the other hand, the largest decrease in hypertension worldwide was seen in high-income super-regions [4], whose results indicate that disparities in the prevalence, treatment, and control of hypertension are expected to occur in different regions.

The average life expectancy of Japanese people was 81.64 years for men and 87.74 years for women in 2020 [6], which suggested that Japan is one of the world’s top countries in terms of longevity [7]. Lifestyle [8], diet [9], and health promotion policies [10] have been related to an increase in the long-life expectancy of the Japanese. In particular, high blood pressure (BP) is the leading cause of the incidence and mortality of CVD in non-communicable diseases (NCDs) in Japan [8,11].

Previous ecological studies have reported that high BP and antihypertensive drug use contributed to the disparity in life expectancy as per prefecture [12]; however, there are no ecological studies that have examined the association between life expectancy and the prevalence, treatment, and control of hypertension over time. We have already reported that lifestyle factors, such as smoking, alcohol consumption, and nutritional intake, were associated with prefectural average life expectancy in cross-sectional and longitudinal ecological studies [13,14]. Therefore, in the present study, we conducted a further ecological study on the long-term changes in prevalence, treatment, and control of hypertension according to groups of prefectures stratified by life expectancy. We used data from the National Health and Nutrition Survey (NHNS) from 1995 to 2016, whose sample is representative of the Japanese population.

The present study aimed to investigate the trends in the prevalence, treatment, and control of hypertension at the prefectural level of life expectancy among Japanese population.

## 2. Materials and Methods

### 2.1. Study Population

The NHNS is an annual cross-sectional household interview and examination survey conducted from October to December (in principle, November) by the Ministry of Health, Labour, and Welfare in Japan. Details of the survey design have been described elsewhere [15,16]. In the present study, we obtained information of 14 years from 1995 to 2016 because the nutrition survey method was changed to a 1-day food weighing method from the 3-day food weighing method in 1995. To examine the overtime trends, the following six terms were used: 1995–1997 (first), 1999–2001 (second), 2003–2005 (third), 2007–2009 (fourth), 2012 (fifth), and 2016 (sixth). The fifth and sixth terms are set as single-year data because the survey scale in 2012 and 2016 was approximately three times larger than usual for the evaluation of the health promotion plan by the Ministry of Health, Labour and Welfare. After excluding people aged <40 or ≥70 years, and participants with missing values when calculating the mean values for each survey item by prefecture, we targeted the following participants: 5086 men and 7426 women in the first term; 4285 men and 6250 women in the second term; 3033 men and 4739 women in the third term; 2982 men and 4380 women in the fourth term; 3262 men and 4895 women in the fifth term; and 2639 men and 3904 women in the sixth term.

This study was conducted using anonymous data from the NHNS obtained through the legal approval of the Ministry of Health, Labour and Welfare. The purpose of the present study and use of NHNS data were applied to the government of Japan, where it was reviewed through due process.

### 2.2. Life Expectancy by Prefecture

According to the life expectancy at the age of 40 by sex in each prefecture in 2000 [17], we classified the prefectures into the following four groups: M1 to M4 for men and F1 to F4 for women, in order of longer life expectancy at the age of 40.

Table 1 shows the life expectancy at the age of 40 years, calculated based on the life tables by prefectures for 1995, 2000, 2005, 2010, and 2015 [17]. The difference between the four groups according to life expectancy at the age of 40 by prefecture in 2000 was almost maintained from 1995 to 2015, including the effects of the Great Hanshin-Awaji Earthquake in 1995.

### 2.3. Measurement and Definition of Hypertension

After the participant had rested for ≥5 min in a sitting position, BP was measured using the Riva-Rocci mercurial sphygmomanometer and the JIS manchette (BP cuff) [18]. Because BP measurements have been taken twice at a time since 2000, we used BP values at the first measurement for the standard comparison. Hypertension was defined as SBP ≥ 140 mmHg and/or diastolic blood pressure (DBP) ≥ 90 mmHg and/or taking of antihypertensive medication. Treatment rate of hypertension was defined as the proportion of those taking of antihypertensive medication among the participants with hypertension. Control rate of hypertension was defined as the proportion of those with SBP < 140 mmHg and DBP < 90 mmHg among participants with hypertension.

### 2.4. Statistical Analyses

Considering both the effects of rapid ageing of the Japanese population and the lower participation rate among younger adults [19], we adjusted for age, based on the 2010 population according to the 10-year age group by sex and prefecture. Furthermore, we calculated age-standardised values for four groups according to the prefectural life expectancy for each term. A two-way analysis of variance was conducted on these age-standardised values based on six terms of annual changes and four groups according to the life expectancy at the age of 40 years. Kumamoto prefecture, where the NHNS was not conducted in 2016, was excluded from the calculation of the mean values for the sixth term.

All statistical analyses were performed using SAS version 9.4 software (SAS Institute, Inc., Cary, NC, USA). All probability values for statistical tests were two-tailed, and *p*-values of <0.05 were regarded as statistically significant.

## 3. Results

The prevalence of hypertension decreased among women in six terms [46.6% (first), 44.5% (second), 43.8% (third), 42.4% (fourth), 41.9% (fifth), and 41.2% (sixth); however, this trend did not occur in men (56.9%, 58.1%, 55.8%, 59.5%, 60.4%, and 59.3%, respectively). The treatment rate of hypertension increased in men (29.5%, 32.8%, 36.8%, 40.8%, 43.9%, and 47.9%) and women (33.5%, 37.1%, 38.7%, 42.9%, 47.6%, and 46.6%). Control rate of hypertension also increased in men (4.6%, 7.2%, 9.1%, 11.0%, 16.5%, and 18.3%) and women (7.7%, 8.7%, 11.1%, 14.8%, 18.7%, and 22.4%).

**Figure 1:**
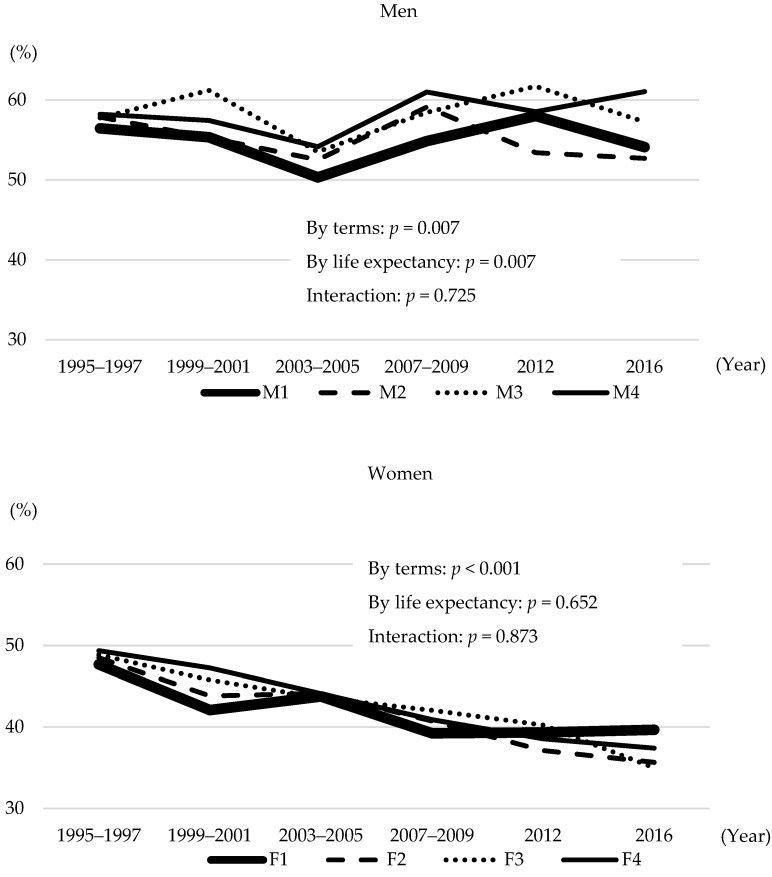
Prevalence of hypertension by four groups according to prefectural life expectancy.

As shown in Figure 1, the age-standardised prevalence of hypertension decreased in both men (*p* = 0.007) and women (*p* < 0.001). There was a significant difference between the age-standardised prevalence of hypertension and life expectancy among men (*p* = 0.007), with M1 remaining at a low value (58.2%, 57.4%, 54.2%, 61.0%, 58.5%, and 61.1% for M4 and 56.5%, 55.4%, 50.3%, 54.9%, 58.0%, and 54.1% for M1, respectively). No significant association was found among women (*p* = 0.652).

**Figure 2:**
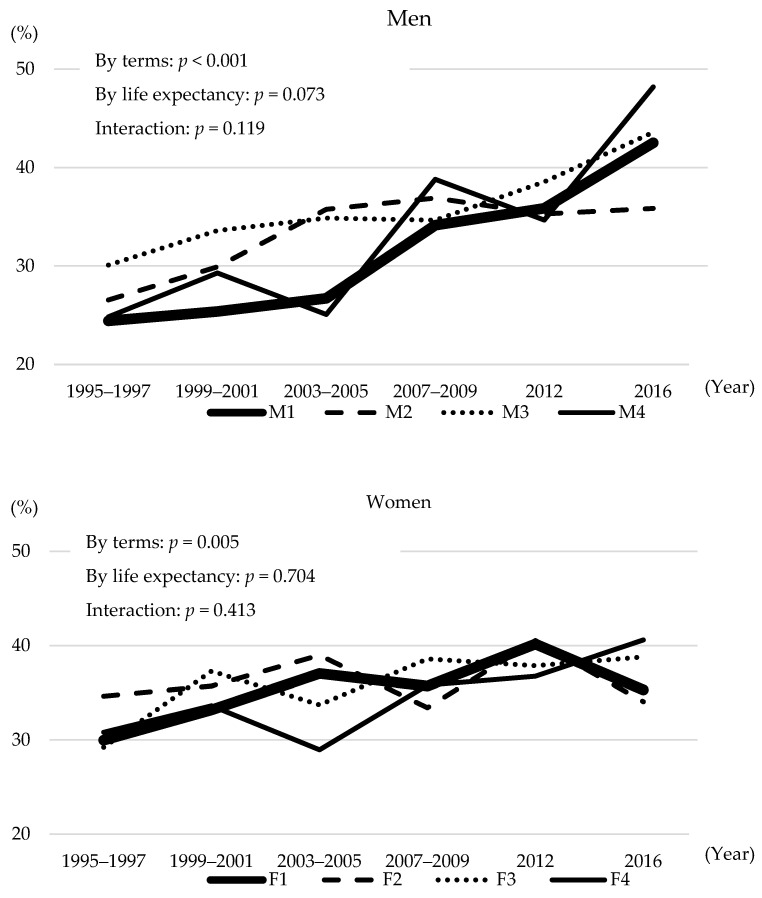
Treatment rate of hypertension by four groups according to prefectural life expectancy.

The age-standardised treatment rate of hypertension by terms increased among both men (*p* < 0.001) and women (*p* = 0.005) (Figure 2). The age-standardised treatment rate of hypertension tended to be associated with life expectancy among men (*p* = 0.073), with M1 remaining at a low value (24.8%, 29.3%, 25.1%, 38.8%, 34.7%, and 48.2% for M4 and 24.4%, 25.4%, 26.7%, 34.1%, 35.9%, and 42.5% for M1, respectively). However, no association was found among women (*p* = 0.704).

**Figure 3:**
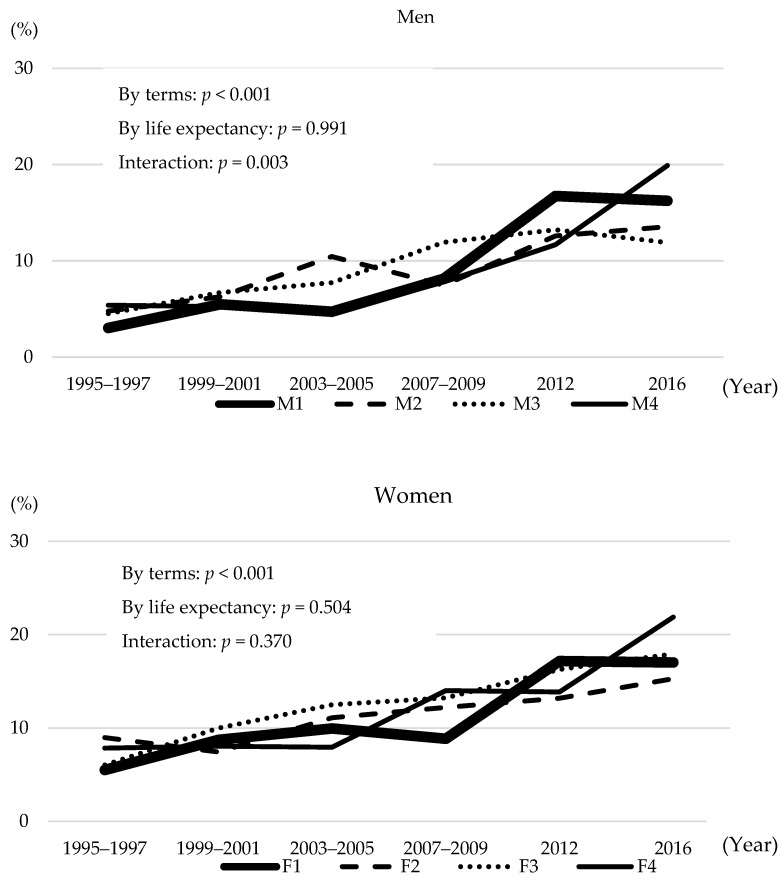
Control rate of hypertension by four groups according to prefectural life expectancy.

The age-standardised control rate of hypertension by terms was increasing among both men (*p* < 0.001) and women (*p* < 0.001); however, there was no significant association between the age-standardised control rate of hypertension and life expectancy among men (*p* = 0.991) and women (*p* = 0.504) (Figure 3).

## 4. Discussion

In the present ecological study on the changes in prevalence, treatment, and control of hypertension during approximately 20 years using the representative data of the Japanese population, we found that the prevalence of hypertension has been decreasing, especially among women, whereas the treatment and control rate of hypertension has been radically increasing in both men and women. The prevalence and treatment of hypertension were significantly associated with life expectancy in each prefecture in men, without significant difference in the control. There were also no significant differences between life expectancy, prevalence, treatment, and control in women.

In Japan, stroke mortality started decreasing in the late 1960s and has been one of the contributors to the longer life expectancy. Hypertension-related stroke deaths have been reported to have a downward trend since the 1980s, and part of this downward trend is related to a decline in overall BP in the population [20]. Potential key factors for the BP decline in the Japanese population are the increased use of antihypertensive drugs among patients with hypertension and a reduction in dietary salt intake [21]. Our previous study reported a significant difference between salt intake and life expectancy among men and women [14]. The present and previous studies support the idea that the hypertension trend or dietary habits contribute to a longer life expectancy.

Previous study reported that the population attributable fractions for CVD death in reference to the optimal BP category tended to be greater for younger groups, accounting for 60.2%, 53.5%, and 13.7% of all CVD deaths in the middle-aged (40–64 years), elderly (65–74 years), and very elderly (75–89 years) groups among men and 59.1%, 43.5%, and 31.5% among women, respectively [22]. According to a previous study from the NHNS and epidemiological studies in Japan [8], tobacco smoking and high BP accounted for 129,000 deaths and 104,000 deaths, respectively, in 2007. They also estimated that controlling SBP and tobacco smoking to optimal counterfactuals would have extended life expectancy at the age of 40 by 1.8 years and 0.9 years among men and 0.9 years and 0.6 years among women, respectively [8]. These results support the findings of our previous [13] and present studies.

A previous ecological study in Japan reported that antihypertensive drug use and SBP were independently and inversely related to life expectancy [12]. In the present study, the association of regional disparities with the prevalence of hypertension among men was observed, in addition to the treatment of hypertension among participants with hypertension in men. In the United States, geographic disparities in life expectancy have been large and increasing between 1980 and 2014, and behavioural and metabolic risk factors (i.e., obesity, inactivity, smoking status, hypertension, and diabetes) have explained 74% of the county-level variation in life expectancy [23]. The Whitehall study, which targeted 19,000 men followed for 38 years, also reported that the difference in life expectancy from age 50 between the highest and the lowest fifths of SBP was 5.2 years [24]. Comparing the findings in the present study with those in other countries, the association between disparities, such as life expectancy and hypertension, seems to be more pronounced in foreign studies. Some of the reasons for this difference could be attributed to the characteristics of the Japanese healthcare system. First, all Japanese adults aged ≥40 years can attend public annual health check-ups at a low cost. A previous study using national health insurance beneficiaries reported that the mortality rates were lower among screeners than non-screeners in Japanese health check-ups [25]. In the Japanese workplace, employers are required to provide annual health check-ups for workers, which may have contributed to the low incidence of coronary artery disease among Japanese workers [26]. Second, the “Kaihoken” system, which was established in Japan in 1961, ensures free access to all types of health services with a self-pay rate of 30% applied across all citizens in Japan. This system has been reported to play an important role in reducing stroke mortality [20].

In the present study, there was no obvious difference in the prevalence and treatment of hypertension according to life expectancy among women. In the Japanese workplace, most full-time workers are men, and health check-ups for full-time workers are mandatory for employers [26]. The participation rate of specific health check-ups, including the employer-obligated ones in Japan, has been higher in men than women aged 40–69 years; however, it has been higher among women than men aged ≥70 years, when the prevalence of the disease increases. Of these, among the individuals insured by municipal national health insurance, the implementation rate has been consistently higher in women than men [27]. The rates of estimated inpatient (per day, per 100,000 population) were higher in men than women, whereas those of outpatients were higher in women than men aged 40–79 years; in particular, the hypertension rates were higher in women than men [28]. Furthermore, women were more concerned about their health and were more likely to attend check-ups and visit medical institutions voluntarily [29]. This may have influenced our results.

However, hypertension causes an economic burden, and grade 3 untreated hypertension has been reported to incur extremely high medical expenditure as a result of hospitalisation [30]. The use of antihypertensive medication may be important not only to prevent serious clinical outcomes which incur extremely high medical costs, but also to solve disparities in life expectancy among prefectures.

In the present study, no association was found between hypertension control and regional disparities. Some people refrain from taking their medications on the day of the survey. In addition, the NHNS is conducted on a date and time when the highest participation can be achieved, considering the circumstances in the national census areas [18]. Therefore, the medication status on the day of BP measurement and the time, including evening and night, of the survey may have affected the results.

The strength of the present study is that it included the participants recruited from community-based census tracts and selected randomly from all over Japan and the use of a standardised method for the BP measurement in each national survey in over 20 years.

This study had limitations. First, hypertension was defined by BP values at a single measurement, which might lead to misclassification of hypertension. However, such misclassification in our study might not have affected the results according to the life expectancy as per prefecture. Second, we used life expectancy at the age of 40 as an overall health indicator for each prefecture; however, we had to limit the target population to those aged 40–69 years. As NCDs, such as CVD, are the major cause of death in Japan and the younger age group has a great impact on life expectancy, the present analysis was conducted on the assumption that hypertension in those aged 40–69 years is related to life expectancy to a certain extent. Furthermore, the previous study reported that hypertension was associated with worse cognitive performance in people aged 50–64 years [31]. According to the report from statistical data in Japan, the prefectures with longer life expectancy tended to be longer healthy life expectancy [32]. However, there are prefectures belonging to a group with a longer life expectancy but with a shorter healthy life expectancy than the national average. Therefore, further studies are needed to examine the association of hypertension with healthy life expectancy. Finally, this was an ecological study based on prefectures over time; thus, note that this study did not evaluate individuals’ BP values and medication status.

## 5. Conclusions

Based on the NHNS data of approximately 20 years, we examined the changes in prevalence, treatment, and control of hypertension according to the life expectancy at the age of 40 years for each prefecture in Japan. The prevalence and treatment of hypertension might have regional disparities according to sex, while the control of hypertension was not significantly related to prefectural disparities according to life expectancy. It may be important to improve lifestyle factors, such as salt intake, thus reducing the hypertension prevalence in each prefecture with a relatively short life expectancy to resolve the disparity in life expectancy.

## Figures and Tables

**Table 1:** Mean life expectancy at the age of 40 years for four groups according to life expectancy by sex in each prefecture in 2000.

Men						Women					
Year	1995	2000	2005	2010	2015	Year	1995	2000	2005	2010	2015
M1	38.84	39.65	40.58	41.34	42.21	F1	44.96	46.26	47.23	47.7	48.12
M2	38.47	39.33	40.27	40.92	41.96	F2	44.43	45.76	46.81	47.31	47.87
M3	38.12	38.97	39.84	40.62	41.58	F3	44.29	45.48	46.53	47.08	47.62
M4	37.68	38.45	39.3	40.09	41.17	F4	43.8	45.14	46.19	46.73	47.32

M1: Kanagawa, Toyama, Fukui, Nagano, Gifu, Shizuoka, Shiga, Kyoto, Nara, Kagawa, Kumamoto, and Okinawa; M2: Miyagi, Yamagata, Gunma, Saitama, Chiba, Tokyo, Ishikawa, Yamanashi, Aichi, Mie, Okayama, and Oita; M3: Hokkaido, Fukushima, Ibaraki, Tochigi, Niigata, Hyogo, Shimane, Hiroshima, Tokushima, Ehime, Nagasaki, and Miyazaki; M4: Aomori, Iwate, Akita, Osaka, Wakayama, Tottori, Yamaguchi, Kochi, Fukuoka, Saga, and Kagoshima; F1: Niigata, Toyama, Ishikawa, Fukui, Yamanashi, Nagano, Shimane, Okayama, Hiroshima, Kumamoto, Miyazaki, and Okinawa; F2: Hokkaido, Miyagi, Shizuoka, Shiga, Kyoto, Nara, Tottori, Kagawa, Kochi, Saga, Nagasaki, and Oita; F3: Iwate, Yamagata, Gunma, Chiba, Kanagawa, Gifu, Mie, Yamaguchi, Tokushima, Ehime, Fukuoka, and Kagoshima; F4: Aomori, Akita, Fukushima, Ibaraki, Tochigi, Saitama, Tokyo, Aichi, Osaka, Hyogo, and Wakayama.

## Data Availability

Data of the National Health and Nutrition Survey is available when the Japanese government gives an approval of application of researchers under regulation of the Statistics Act.
